# Exploring the first Rimonabant analog-opioid peptide hybrid compound, as bivalent ligand for CB1 and opioid receptors

**DOI:** 10.1080/14756366.2016.1260565

**Published:** 2017-01-18

**Authors:** Adriano Mollica, Sveva Pelliccia, Valeria Famiglini, Azzurra Stefanucci, Giorgia Macedonio, Annalisa Chiavaroli, Giustino Orlando, Luigi Brunetti, Claudio Ferrante, Stefano Pieretti, Ettore Novellino, Sandor Benyhe, Ferenc Zador, Anna Erdei, Edina Szucs, Reza Samavati, Szabolcs Dvorácskó, Csaba Tomboly, Rino Ragno, Alexandros Patsilinakos, Romano Silvestri

**Affiliations:** a Dipartimento di Farmacia, Università di Chieti-Pescara “G. d’Annunzio”, Chieti, Italy;; b Dipartimento di Chimica e Tecnologie del Farmaco, Istituto Pasteur Italia-Fondazione Cenci Bolognetti, Sapienza Università di Roma, Roma, Italy;; c Dipartimento del Farmaco, Istituto Superiore di Sanità, Rome, Italy;; d Dipartimento di Farmacia, Università di Napoli “Federico II”, Naples, Italy;; e Institute of Biochemistry, Biological Research Centre, Hungarian Academy of Sciences, Szeged, Hungary;; f Dipartimento di Chimica e Tecnologie del Farmaco, Rome Center for Molecular Design, Sapienza Università di Roma, Roma, Italy;; g Alchemical Dynamics s.r.l, Roma, Italy

**Keywords:** Cannabinoid receptor CB1R, Rimonabant, opioids, bivalent ligand, pain

## Abstract

Cannabinoid (CB) and opioid systems are both involved in analgesia, food intake, mood and behavior. Due to the co-localization of µ-opioid (MOR) and CB1 receptors in various regions of the central nervous system (CNS) and their ability to form heterodimers, bivalent ligands targeting to both these systems may be good candidates to investigate the existence of possible cross-talking or synergistic effects, also at sub-effective doses. In this work, we selected from a small series of new Rimonabant analogs one CB1R reverse agonist to be conjugated to the opioid fragment Tyr-D-Ala-Gly-Phe-NH_2_. The bivalent compound (**9**) has been used for *in vitro* binding assays, for *in vivo* antinociception models and *in vitro* hypothalamic perfusion test, to evaluate the neurotransmitters release.

## Introduction

Cannabinoid and opioid receptors are expressed mostly in the same CNS areas, and both are involved in the control of analgesia, food intake, mood and behavior. In the dorsal horn of the spinal cord, the µ-opioid receptor MOR and CB1R are co-localized at the same neurons, also at the supra-spinal level, such as the periaqueductal gray (PAG), the raphe nuclei and the central-medial thalamic nuclei^1^.

According to the several cell line studies where the MOR and CBR1 are endogenously co-expressed, they share cAMP signaling pathways, even though they may use different sets of G-proteins^2^.

Studies on opioid and CB1R knock-out mice, demonstrated that their density and activity strongly depend on each other^3^^,^ 4. Also, preclinical and clinical studies stated that the interaction between the opioid and cannabinoid systems can lead to promising therapeutic applications in pain control and in alimentary disorders management^5–8^. Thus, bivalent ligands binding to CB1R and MOR simultaneously may result in new potent analgesic agents. Recently, Le Naour et al.^6^ proposed a bivalent approach to target both MOR and CB1R, connecting a selective MOR agonist to a CB1R selective inverse agonist, *via* a spacer group of varied length.

One of the synthesized compounds showed an extremely potent activity in *in vivo* antinociceptive tests and was devoid of tolerance. Additive or synergic interactions between opioid and cannabinoid systems in producing analgesia have been previously described and reviewed in detail^9^^,^ 10. Morphine-induced antinociception is completely reversed by the CB1R antagonist AM251^11^, and tetrahydrocannabinol (THC)-induced antinociception is blocked by naloxone^12^.

The cross-tolerance between THC and morphine and the possibility that these receptors interact pharmacologically were demonstrated by naloxone or CB1R antagonist. Synergism between cannabinoids and opioids at sub-effective doses has also been reported^13^^,^ 14. Additionally, co-administration of morphine with a CB1R antagonist inhibited the development of both acute and chronic tolerance to morphine^15^.

Other evidence for interaction was obtained from self-administration studies showing that both receptors are involved in reward processes. In this regard, both CB1R antagonist (SR 141716)^16^, and opioid antagonist naloxone decreased self-administration of morphine or Δ^9^-THC^17^. In mice lacking µ (MOR) and δ (DOR) opioid receptors, the cannabinoid withdrawal syndrome is reduced^18^.

According to our research line based on the design of multitarget compounds^19^^,^ 20, the present study represents a starting point to develop bivalent ligands as pharmacologic tools to investigate the MOR–CB1R mutual interactions. The bivalent ligand designed for this purpose consists of a selective µ-receptor peptide agonist connected to a CB1R selective inverse agonist fused together^21^.

The novel compound was extensively tested for *in vitro* binding, GTP stimulation, neurotransmitters release and antinociceptive *in vivo* activity. The design rationale for targeting MOR and CB1R simultaneously is based on previous studies with bivalent ligands^6^. The MOR agonist pharmacophore derived from the opioid peptide biphalin, was previously employed in the design of several bivalent compounds, due to its property to well tolerate the connection at the *C*-terminus with another pharmacophore^22^^,^ 23 ^,^ 19.

**Figure 1.**
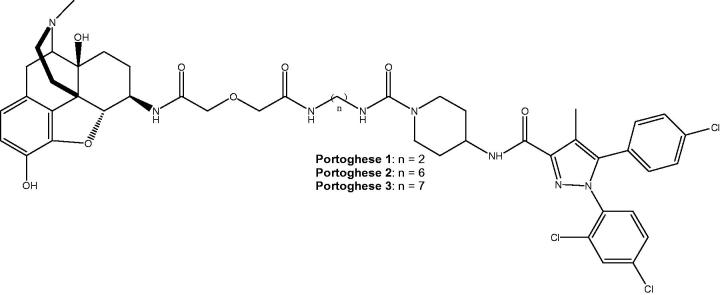
Structure of bivalent compounds previously designed by Le Naour et al.^6^

**Scheme 1.**
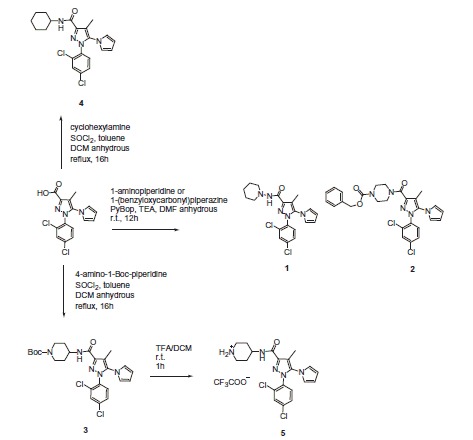
Synthesized Rimonabant analogs.

Since other authors show that a CB1R inverse agonist is capable of eliminating morphine tolerance and dependence^24^^,^ 25, we selected compound **5** (Scheme 1) with CB1R inverse agonist activity as CB1R pharmacophore (Figure 1), in analogy with the work by Le Naour et al.^6^

Thus, the bivalent ligand **9** was synthesized following the “fused-bivalent approach”^21^, with the expectation that the resulting product would be capable to interact at CB1R, MOR and CB1R–MOR heterodimer form. The bivalent compound was prepared by coupling each Boc-protected amino acid of the opioid peptide sequence to the secondary amine of 4-aminopiperidine group present in the structure of Rimonabant analog **5**.

The opioid fragment Tyr-D-Ala-Gly-Phe-NH_2_ (**10**) was used as reference compound for opioid activity on MOR and DOR^19^.

## Results and discussion

A series of novel 1-aryl-5-(1H-pyrrol-1-yl)-1H-pyrazole-3-carboxamides Rimonabant analogs were tested for the stimulation of G protein to evaluate their inverse agonist activity at CB1R to develop a bivalent compound (Scheme 1).

**Figure 2.**
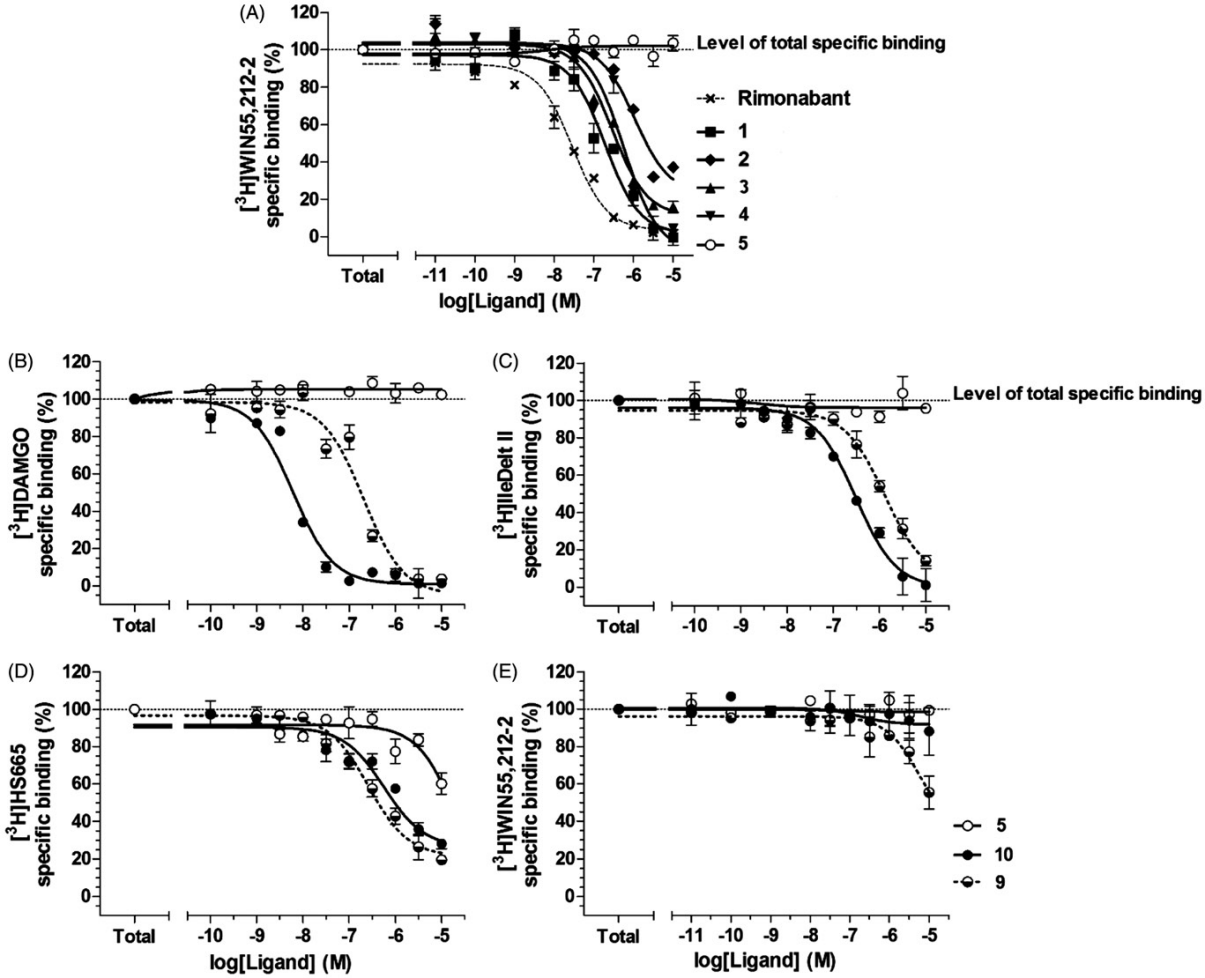
The binding affinity of Rimonabant and its analogs on CBR (A) and the MOR (B), DOR (C), KOR (D) and CBR binding affinity of **5**, **9** and **10** (Tyr-D-Ala-Gly-Phe-NH_2_) (E) in competition binding experiments. Figures represent the specific binding of [^3^H]WIN55 212–2, [^3^H]DAMGO, [^3^H]IleDelt II and [^3^H]HS665 in percentage in the presence of increasing concentrations (10 ^−11^–10^−5^ M) of the indicated unlabeled ligands performed in rat (A, B and E) or in guinea pig (D) whole brain membrane homogenates. “Total” on the *x*-axis indicates the total specific binding of radioligand, which is measured in the absence of the unlabeled compounds. The level of total specific binding was defined as 100% and is presented with a dotted line. Points represent means ± SEM for at least three experiments performed in duplicates.

Compounds **1** and **2** have been previously synthesized and characterized by Silvestri et al.^26^ for the development of potent CBR1 inverse agonists. The molecules were designed by pursuing a bioisosteric approach on Rimonabant, from which **5** resulted to be one of the most interesting candidate with the advantage to be easily derivatisable by coupling with an opioid peptide on the piperidine secondary nitrogen in place of the *tert*-butoxycarbonyl (Boc) group. Indeed *N*-Boc derivative **3** resulted to be active, showing that the substitution of a bulky moiety at the secondary nitrogen of the 4-aminopiperidine terminus was possible without any loss of activity. Firstly, we investigated the cannabinoid receptor (CBR) binding affinity of the Rimonabant analogs compared with Rimonabant in competition binding assays using the nonselective cannabinoid receptor radioligand **[** ^3^H]WIN55 212 (CB1R < CB2R) (Figure 2). In this test, the Rimonabant analogs **1–4** exhibited nanomolar Ki values: compounds **1** (Ki =125.9 nM) and **3** (Ki =192.9 nM) were the most potent derivatives as compared with Rimonabant (Ki =25 nM) (Table S1, see SI)^27^^,^ 28.

In the following step, we investigated the effect of Rimonabant and its analogs on G-protein activity. In these experiments, we determined their agonist, antagonist or inverse agonist nature. The experiments were performed by [35S]GTPγS binding assays to control the GDP to GTP exchange of the α-subunit of G_i_-protein. The analogs **1**, **2**, **3** and **4** decreased [35S]GTPγS specific binding (thus G-protein activity) compared with basal activity by more than 80% (E_max _= 17.5). Compounds **1–4** showed strong reduction of G-protein activity comparable to Rimonabant^29–31^ and therefore displayed inverse agonist effects (Table 1). We examined the opioid receptor binding affinity of **5** in competition binding experiments, using opioid receptor selective radioligands. As expected, **5** did not bind to MOR and DOR and the affinity for the KOR was negligible (Table 1S, Figure 2(B–D))^31–37^.

**Table 1.** The maximal G-protein efficacy (E_max_) and ligand potency (logEC_50_) of the Rimonabant and its analogs **1**, **2**, **3**, **4**, **5**, bivalent compound **9** and opioid peptide **10** in [^35^S]GTPγS binding assays on rat brain membrane homogenates. The values were calculated according to dose–response curves in Figure S1 (see SI) as described in the “Data analysis” section.

	E_max_ ± S.E.M. (%)	LogEC_50_ ± S.E.M. (EC_50_)
Rimonabant	17.5 ± 5.72	−5.65 ± 0.12 (2.2 μM)
1	26 ± 5.27	−5.78 ± 0.11 (1.62 μM)
2	22.95 ± 4.3	−5.81 ± 0.09 (1.54 μM)
3	12.78 ± 8.48	−5.57 ± 0.15 (2.69 μM)
4	55.95 ± 8.44	−5.72 ± 0.3 (1.87 μM)
5	99.2 ± 46.1	ambiguous*
Bivalent compound (**9**)	36.62 ± 10.35	−5.44 ± 0.21 (3.6 μM)
Tyr-D-Ala-Gly-Phe-NH_2_ (**10**)	161.5 ± 4.08	−7.09 ± 0.22 (81.3 nM)

* Since the compound did not alter significantly the total specific binding of the radioligand, thus logEC_50_ values cannot be interpreted.

Opioid fragment **10** displayed a high affinity for MOR and DOR (MOR > DOR), while the compound had modest affinity for KOR, even at high nanomolar concentration (Table S1). As expected, the opioid peptide fragment did not show any affinity for CBR1 (Table 1S, Figure 2(E)); it increased G-protein activity with a maximum efficacy (E_max_) of nearly 70% above basal activity with EC_50_ of 81.3 nM, thus we can assert that this compound behaved as an agonist.

**Scheme 2.**
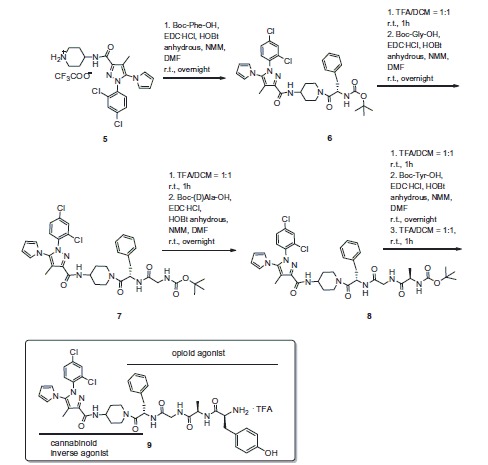
Synthesis of bivalent compound **9**.

In the second part of our work, the designed bivalent compound **9** was synthesized (Scheme 2) and fully characterized (see SI). Peptide **10** has been prepared following the standard synthetic procedure for coupling reactions^19^.

Compound **9** behaved as inverse agonist as it decreased [35S]GTPγS specific binding, thus reducing G-protein activity compared with the basal level (Table 1). Attaching the opioid fragment to Rimonabant analog **5** to give the bivalent compound **9** resulted in a rather selective DOR ligand with an unexpected improvement of affinity for KOR, and a loss in affinity for MOR and DOR, as compared with the opioid fragment alone (Table S1, Figure 2(B–D)). According to the affinities displayed by the opioid peptide **10** and **5**, the bivalent compound **9** showed modest affinity towards CB1R (Table S1, Figure 2(E)).

**Figure 3.**
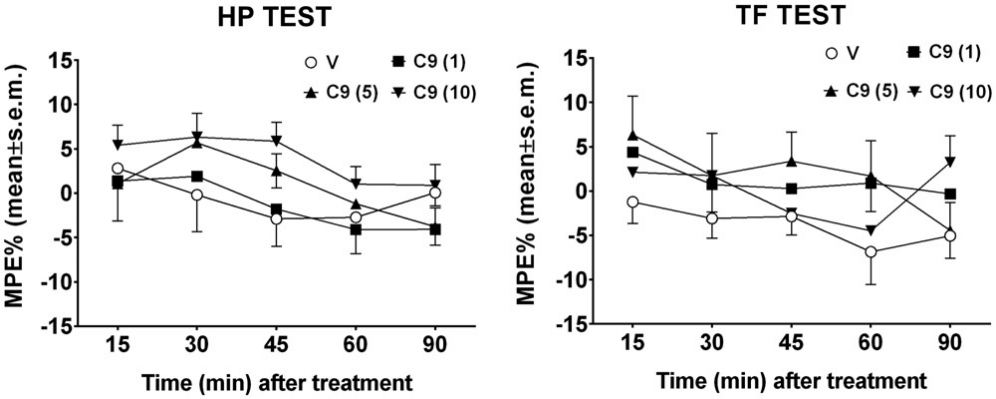
Hot-plate and Tail flick test. In these experiments, compound **9** (C9) was administered i.c.v. at doses of 1, 5 and 10 μg/mouse. V is for vehicle. *N* = 10.

The results obtained in the hot plate and tail flick tests after i.c.v. injection in mice, are reported in Figure 3. In these experiments, compound **9** was administered i.c.v. at doses of 1, 5 and 10 µg/mouse. Neither in the hot plate nor in the tail flick test treatments modified the behavioral response to thermal nociceptive stimuli. In the hot plate test, two-way ANOVA revealed no difference in treatments [*F*_3,120 _=_ _2.65, *p* = 0.522] and in time [*F*_4,120 _=_ _2.30, *p* = 0.0629] paradigms. Similar absence of effect was observed in the tail flick test, since treatment [*F*_3,120 _=_ _2.6, *p* = 0.0551] did not affect the latency of the nociceptive response all over the time [*F*_4,120 _=_ _1.32, *p* = 0.2649].

**Figure 4.**
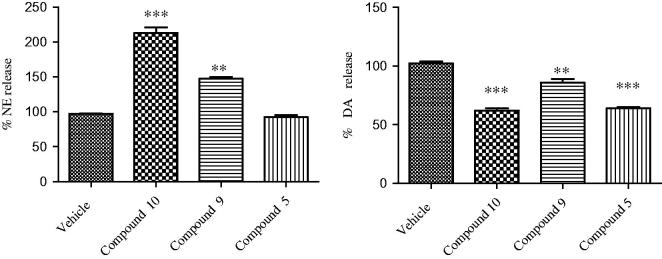
Effect of opioid peptide **10**_,_ bivalent compound **9** and **5** on NE and DA release from hypothalamic synaptosomes, *in vitro*. ANOVA *p* < 0.0001, ***p < 0.001, **p < 0.01 vs. vehicle.

**Figure 5.**
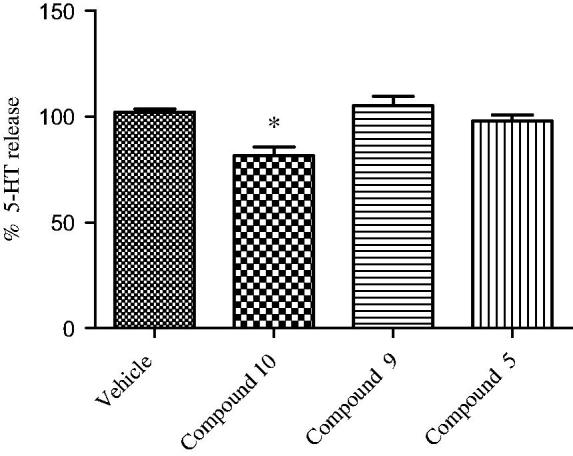
Effect of opioid peptide **10**, bivalent compound **9** and **5** on 5-HT release from hypothalamic synaptosomes, *in vitro*. ANOVA *p* < 0.05, *p < 0.05 vs. vehicle.

To further explore the pharmacologic profile of the bivalent ligand, hypothalamic perfusion test was performed with the aim to compare the release of hypothalamic neurotransmitters, after administration of the two separate pharmacophores and compound **9** in the preparation of synaptosomes. In this experiment, the three combinations produced different effects, showing a possible influence of the contemporary stimulation of CBRs and opioid systems on neurotransmitters release. As regards to **10**, we found a significant stimulatory effect on norepinephrine (NE) and an inhibitory effect on serotonin (5-HT) and dopamine (DA) release, from hypothalamic synaptosomes (Figures 4 and 5).

Administration of compound **5** decreased only the DA release, whereas the treatment with **9** resulted in the reduction of the NE release’s stimulation and DA inhibition (in less extent), with no significant effects on the 5-HT release. This is consistent with the inverse agonist activity on the G-protein system of compound **9**. The significant stimulatory effect on NE release could indicate a possible involvement in the regulation of energy balance. By contrast, the reduced inhibitory effect on hypothalamic DA release, compared with both parent molecules, is coherent with a minor potential of behavioral adverse effects *in vivo* 38 ^,^ 39.

The modulatory effects on hypothalamic biogenic amines suggest a possible involvement of both **10** and **5** in the interconnected neuronal pathways for the energy balance control. 5-HT release plays an anorectic role in the hypothalamus^40^; DA injection in the perifornical hypothalamus inhibited food intake^41^, while DA administration in the lateral hypothalamus stimulated feeding^42^. NE could also exert both anorexigenic and orexigenic effects, related to the activation of α1- and α2-adrenoceptors, respectively^43^. In addition, NE binding to hypothalamic α2-adrenoceptors could increase energy expenditure, through the stimulated sympathetic activity^44^. Previously, we observed that endomorphin-2 (EM-2), a selective MOR agonist, was able to stimulate food intake^45^. The orexigenic effect was, albeit partially, related to the stimulated hypothalamic DA and NE activity which is also consistent with the increased oxygen consumption induced by EM-2 administration to mice^46^.

The Absorption, Distribution, Metabolism, Excretion and Toxicity (ADMET) properties of compounds **5, 9** and **10** were assessed by means of web servers and specialized programs. Several tools are available to profile compounds ADMET properties using *in silico* calculations. In Tables 2S–4S are reported the admetSAR^47^, Molinspiration and cbligand.org ADMET calculated properties. In general, compounds **5**, **9** and **10** are not substrate for cytochromes and present low toxicity profiles (Table 2).

**Table 2.** AdmetSAR regression derived data for diverse chemicals associated with known ADMET profiles for compounds **5**, **9** and **10**.

	Model	Unit	5	9	10
Absorption	Aqueous solubility	LogS	−3.0087	−3.1063	−3.3561
	Caco-2 permeability	LogP_app_, cm/s	0.4939	0.1530	−0.2667
Toxicity	Rat acute toxicity	LD_50_, mol/kg	2.6224	2.6993	2.2387
	Fish toxicity	pLC_50_, mg/L	1.6053	1.3864	1.6515
	Tetrahymena pyriformis toxicity	pIGC_50_, μg/L	0.5156	0.4875	−0.1386

Regarding adsorption, derivative **9** violates three Lipinski’s rules (high MW, number of HB donators and acceptors) that could prevent its oral bioavailability. AdmetSAR BBB and CACO predicted permeability indicate compound **5** as likely permeable, while compounds **9** and **10** are predicted at low probability to permeate BBB and CACO cells. These values are somehow in agreement with those reported in Table 4S where eight QSAR models indicate **5** as fully able to penetrate BBB, while **9** and **10** are predicted on the edge among positive and negative BBB penetrating molecules, as evinced from plots and threshold values reported in Table 5S.

To further inspect on possible BBB permeability, new models were herein developed by means of the python programming language, open-source cheminformatics library rdkit (www.rdkit.org), mathematical and scientific libraries numpy and scipy, machine-learning library scikit-learn^48^. Application of the two new models confirmed that compound **5** is able to penetrate BBB, while compounds **9** and **10**, although predicted not able to penetrate BBB, show some probability to cross the BBB. The graphical analyses of similarity maps (Figure 2S) indicate the molecular portions likely responsible for the positive/negative BBB penetration^49^.

According to our experiments, we can conclude that the receptor binding profile and biological activity of the bivalent compound **9** significantly changed compared with the individual components. The bivalent compound **9** showed higher selectivity for MOR than the enkephalin-like opioid peptide fragment **10**. More interestingly, it showed an improved KOR affinity compared with **5** and **10**. The stimulation of the G-protein system could be explained by the inverse agonist effect of the bivalent compound. The lack of antinociceptive effect deals with the biological profile of an inverse agonist triggering the G-protein cascade. Also the ADMET properties of **9** are critical and establish benchmarks for further development of this class of bivalent compounds. Further studies on an alternative design of novel bivalent compounds based on an opioid peptide and a Rimonabant analog are currently undergoing in our laboratory.

## Supplementary Material

IENZ_1260565_Supplementary_Material.pdf
