# Supplementary material for: Exploring the first Rimonabant analog-opioid peptide hybrid compound, as bivalent ligand for CB1 and opioid receptors
Source: J Enzyme Inhib Med Chem. 2017 Jan 18;32(1):444–51. doi: 10.1080/14756366.2016.1260565 (PMC6009935; doi:10.1080/14756366.2016.1260565)
Supplement: IENZ_1260565_Supplementary_Material.pdf [file IENZ_A_1260565_SM4429.pdf]

Exploring the first Rimonabant analogue-opioid peptide hybrid compound, as bivalent ligand for CB1 and opioid receptors

Adriano Mollica,[1] Sveva Pelliccia,[2] Valeria Famiglini,[2] Azzurra Stefanucci,[1] Giorgia Macedonio,[1] Annalisa Chiavaroli,[1] Giustino Orlando,[1] Luigi Brunetti,[1] Claudio Ferrante,[1] Stefano Pieretti,[3] Ettore Novellino,[4] Benyhe Sandor,[5] Ferenc Zador,[5] Anna Erdei, [5] Edina Szucs,[5] Reza Samavati,[5] Szalbolch Dvrorasko,[5] Csaba Tomboly,[5] Rino Ragno,[6,7] Alexandros Patsilinakos,[6,7] Romano Silvestri. [2,*]

[1] Dipartimento di Farmacia, Università di Chieti-Pescara "G. d'Annunzio", Via dei Vestini 31, 66100 Chieti, Italy.
[2] Istituto Pasteur Italia-Fondazione Cenci Bolognetti, Dipartimento di Chimica e Tecnologie del Farmaco, Sapienza Università di Roma, Piazzale Aldo Moro 5, I-00185 Roma, Italy.
[3] Istituto Superiore di Sanità, Dipartimento del Farmaco, Viale Regina Elena 299, 00161, Rome, Italy.
[4] Dipartimento di Farmacia, Università di Napoli "Federico II", Via D. Montesano, 49, 80131 Naples, Italy.
[5] Institute of Biochemistry, Biological Research Centre, Hungarian Academy of Sciences, 6726, Szeged, Hungary.
[6] Rome Center for Molecular Design, Dipartimento di Chimica e Tecnologie del Farmaco, Sapienza Università di Roma, P. le A. Moro 5, 00185 Roma, Italy.
[7] Alchemical Dynamics s.r.l., 00125 Roma, Italy.

*Address correspondence to this author at the Dipartimento di Chimica e Tecnologie del Farmaco, Sapienza Università di Roma, Piazzale Aldo Moro 5, I-00185 Roma, Italy; E-mail: romano.silvestri@uniroma1.it

TABLE OF CONTENTS

EXPERIMENTAL SECTION<br>METHODS AND MATERIALS<br>CHEMISTRY	PAGS. 2-6
IN VITRO BINDING ASSAYS	PAGS. 7-10
Table 1S	PAG. 9
Figure 1S	PAG. 10
IN VIVO NOCICEPTION TESTS	PAG. 11
IN VITRO HYPOTHALAMIC PERFUSION TEST	PAG. 12
ADMET *in silico* profiling	PAGS. 13-17
Table 2S	PAG. 14
Table 3S	PAG. 14
Table 4S	PAG. 15
Table 5S	PAG. 16
Table 6S	PAG. 16
Figure 2S	PAG. 17
REFERENCES	PAG. 18,19

EXPERIMENTAL SECTION
Methods and Materials

All reagents and solvents are commercially available and used without further purification. All amino acids were purchased from Fluka. Solution phase peptide synthesis was performed on the basis of the standard protocol using Boc-protected amino acids. Organic solutions were dried over anhydrous sodium sulfate. Evaporation of the solvents was carried out on a Büchi Rotavapor R-210 equipped with a Büchi V-850 vacuum controller and Büchi V-700 (~5 mbar) and V-710 (~2 mbar) vacuum pumps. Column chromatography was run on glass columns packed with alumina (Merck, 70-230 mesh) or silica gel (Macherey-Nagel, 70-230 mesh) eluting with the indicated solvent. Aluminium oxide thin layer chromatography (TLC) cards from Fluka (aluminium oxide precoated aluminium cards with fluorescent indicator visualizable at 254 nm) and silica gel TLC cards from Macherey-Nagel (silica gel precoated aluminum cards with fluorescent indicator visualizable at 254 nm) were used for TLC. Developed plates were visualized by a Spectroline ENF 260C/FE UV apparatus. Melting points (mp) were determined on a SMP1 apparatus (Stuart Scientific) and are uncorrected. IR spectra were run on a SpectrumOne FT-ATR spectrophotometer (Perkin Elmer). Band position and absorption ranges are given in cm^{-1}. Proton (1H NMR) nuclear magnetic resonance spectra were recorded on a 300 MHz FT spectrometer (Bruker) in the indicated solvent. Chemical shifts are expressed in δ units (ppm) from tetramethylsilane. Elemental analyses of tested compounds were found within 0.4% of the theoretical values. Combustion analysis was used as a method of establishing compound's purity. Purity of tested compounds was ≥95%. Mass spectral (MS) data were obtained using Agilent 1100 LC/MSD VL system (G1946C) with a 0.4 mL/min flow rate using a binary solvent system of 95:5 methanol/water. UV detection was monitored at 230, 254, 275 nm.

Chemistry

Pyrazolecarboxamides **1** and **2** were prepared by reaction of 1-(2,4-dichlorophenyl)-4-methyl-5-(1*H*-pyrrol-1-yl)-1*H*-pyrazole-3-carboxylic acid with the appropriate amines in the presence of PyBop reagent and triethylamine in anhydrous DMF at 25°C for 12 hours, as previously described.[1]

Pyrazolecarboxamides **3** and **4** were obtained by reaction of 1-(2,4-dichlorophenyl)-4-methyl-5-(1*H*-pyrrol-1-yl)-1*H*-pyrazole-3-carboxylic acid with thionyl chloride in anhydrous toluene at reflux temperature for 3 hours and subsequent treatment with appropriate amines in anhydrous dichloromethane for 16 hours. Compound **5** was obtained by reaction of **3** with trifluoroacetic acid in anhydrous dichloromethane at 25°C for 1 hour.

Preparation of *Tert*-butyl-4-(1-(2,4-dichlorophenyl)-4-methyl-5-(1*H*-pyrrol-1-yl)-1*H*-pyrazole-3-carboxamido)piperidine-1-carboxylate 3. A mixture of 1-(2,4-dichlorophenyl)-4-methyl-5-(1*H*-pyrrol-1-yl)-1*H*-pyrazole-3-carboxylic acid (50 mg, 0.15 mmol) and thionyl chloride (0.039 mL, 0.54 mmol) in 1.5 mL of toluene was refluxed for 3 hours and then evaporated to dryness under reduced pressure. The residue was taken up in 1mL of toluene and solvent was evaporated again under reduced pressure to give the crude acid chloride, which was dissolved in 1.5 mL of dry dichloromethane. This solution was added dropwise to a solution of 4-amino-1-Boc-piperidine (44 mg, 0.22 mmol) in 1.0 mL of dichloromethane cooled to 0°C. After being stirred at room temperature for 16 hours, the reaction mixture was added to brine and extracted with dichloromethane. Organic layer was dried and filtered. Removal of the solvent gave a residue that was purified by silica gel column chromatography (ethyl acetate/*n*-hexane = 1:3 as eluent) to furnish **3** in 89% yield as a white solid (mp 115-120°C from ethanol).

Preparation of *N*-cyclohexyl-1-(2,4-dichlorophenyl)-4-methyl-5-(1*H*-pyrrol-1-yl)-1*H*-pyrazole-3-carboxamide 4. A mixture of 1-(2,4-dichlorophenyl)-4-methyl-5-(1*H*-pyrrol-1-yl)-1*H*-pyrazole-3-carboxylic acid (50 mg, 0.15 mmol) and thionyl chloride (0.039 mL, 0.54 mmol) in 1.5 mL of toluene was refluxed for 3 hours and then evaporated to dryness under reduced pressure. The residue was taken up in 1mL of toluene and solvent was evaporated again under reduced pressure to give the crude acid chloride, which was dissolved in 1.5 mL of dry dichloromethane. This solution was added dropwise to a solution of cyclohexylamine (22 mg, 0.025 mL, 0.22 mmol) in 1.0 mL of dichloromethane cooled to 0°C. After being stirred at room temperature for 16 hours, the reaction mixture was added to brine and extracted with dichloromethane. Organic layer was dried and filtered. Removal of the solvent gave a residue that was purified by silica gel column chromatography (ethyl acetate/*n*-hexane = 1:3 as eluent) to furnish **4** in 75% yield as a white solid (mp 180-187°C from ethanol).

^{1}H-NMR (DMSO-*d6*): δ 1.03-1.13 (m, 1H), 1.22-1.40 (m, 4H), 1.56-1.59 (m, 1H), 1.68-1.76 (m, 4H), 2.12 (s, 3H) 3.72-3.79 (m, 1H), 6.14 (s, 2H), 6.78 (s, 2H), 7.56 (d, *J* = 8.7 Hz, 1H), 7.73 (d, *J* = 8.3 Hz, 1H), 7.80 (s, 1H), 7.98 (d, *J* = 8.8 Hz, 1H, disappeared on treatment with D_2O), IR: ν 1662, 2931, 3407 cm^{-1}. Anal. Calcd. for ($C_{21}H_{22}Cl_2N_4O$ (417,33)) C, 60.44%; H, 5.31%; N, 13.43%; Cl, 16.99%. Found: C, 60.53%; H, 5.34%; N, 13.39%; Cl, 16.94%.

Preparation of 4-(1-(2,4-dichlorophenyl)-4-methyl-5-(1*H*-pyrrol-1-yl)-1*H*-pyrazole-3-carboxamido)piperidin-1-ium 2,2,2 trifluoroacetate 5. Compound **3** (76 mg, 0.15 mmol) was taken up in dichloromethane (2 mL) and trifluoroacetic acid was added (20% v/v).The mixture was stirred at 25°C for 1 hour. The solvent was then evaporated under reduced pressure and the residue was co-evaporated with diethyl ether to give **5** in 57% yield as white powder (mp 100-105°C from ethanol).

^{1}H-NMR (DMSO-*d6*): δ 1.71-1.81 (m, 2H), 1.87-1.92 (m, 2H), 2.13 (s,3H), 2.95-3.04 (m, 2H), 3.28-3.31 (m, 2H), 4.03-4.10 (m, 1H), 6.14 (t, *J* = 2.0 Hz, 2H), 6.78 (t, *J* = 2.2 Hz, 2H), 7.57 (dd, *J* = 2.3 and *J* = 8.5 Hz, 1H), 7.74-7.76 (m, 1H), 7.82 (d, *J* = 2.3 Hz, 1H), 8.19-8.27 (m, 1H, disappeared on treatment with D_2O), 8.48 (d, *J* = 8.0 Hz, 1H, disappeared on treatment with D_2O), 8.53-8.57 (m, 1H, disappeared on treatment with D_2O), IR: ν 1682, 2967 cm^{-1}. Anal. Calcd. for ($C_{22}H_{22}Cl_2F_3N_5O_3$ (532,34)) C, 49.64%; H, 4.17%; N, 13.16%; Cl, 13.32%; F, 10.71% Found: C, 49.69%; H, 4.67%; N, 13.29%; Cl, 13.34%; F, 10.80%.

Preparation of the intermediate compound 6. To a stirred solution of **5** (1 equiv., 60 mg) in DMF (6 mL) at 0°C, EDC (1.1 equiv., 24 mg) and HOBt anhydrous (1.1 equiv., 19 mg) were added. After 10 min., NMM (1.1 equiv., 14 μL) and Boc-Phe-OH (1.1 equiv., 33 mg) were dissolved in the reaction mixture and stirred at 0°C for 10 min. After 24 hours at r.t., the reaction mixture was evaporated in rotary evaporator, the residue was dissolved in EtOAc and washed with 5% citric acid solution (3 times), $NaHCO_3$ s.s. (3 times) and NaCl s.s. (3 times). The organic layers were collected and dried over Na_2SO_4 anhydrous and filtered. The organic solvent was removed in rotary evaporator and high vacuum to obtain the crude product in 90% yield (eluent: EtOAc/DCM = 7:3; R*f* = 0.8). The intermediate compound was treated with a mixture of TFA/DCM = 1:1 at room

temperature for 1h, then was used as TFA salt for the following reaction without further modification.

^{1}H-NMR (300 MHz, $CDCl_3$) δ: 7.51 (d, 1H), 7.45 (d, 1H), 7.32-7.15 (m, 6H), 6.77 (d, 1H), 6.56 (m, 2H), 6.19 (m, 2H), 5.44 (d, 1H), 4.06 (m, 1H), 2.98 (q, 2H), 2.69 (m, 4H), 2.27 (s, 3H), 1.92 (m, 1H), 1.69 (m, 4H), 1.40 (s, 9H). LRMS (ESI) m/z 780.5 $[M+H]^+$.

Preparation of intermediate compound 7. To a stirred solution of Boc-Gly-OH (1 equiv., 17 mg) in DMF (6 mL) at 0°C, EDC (1.1 equiv., 19 mg) and HOBt anhydrous (1.1 equiv., 16 mg) were added. After 10 min., NMM (1.1 equiv., 11 μL) and **6** as TFA salt (1.1 equiv.), were dissolved in the reaction mixture and stirred at 0°C for 10 min. After 24 hours at r.t., the reaction mixture was evaporated in rotary evaporator, the residue was dissolved in EtOAc and washed with 5% citric acid solution (3 times), $NaHCO_3$ s.s. (3 times) and NaCl s.s. (3 times). The organic layers were collected and dried over Na_2SO_4 anhydrous and filtered. The organic solvent was removed in rotary evaporator and high vacuum to obtain the crude product in 95% yield (eluent: EtOAc/MeOH = 9:1, R*f* = 0.3). The intermediate compound was treated with a mixture of TFA/DCM = 1:1 at room temperature for 1h, then was used as TFA salt for the following reaction without any further modification.

^{1}H-NMR (300 MHz, $CDCl_3$) δ: 7.51 (d, 1H), 7.45 (d, 1H), 7.32-7.15 (m, 6H), 7.02 (d, 1H), 6.77 (d, 1H), 6.56 (m, 2H), 6.19 (m, 2H), 5.14 (t, 1H), 4.45 (m, 2H), 3.80 (t, 2H), 2.98 (q, 4H), 2.68 (m, 1H), 2.26 (s, 3H), 1.69 (m, 4H), 1.40 (s, 9H). LRMS (ESI) m/z 837.6 $[M+H]^+$.

Preparation of intermediate compound 8. To a stirred solution of Boc-(D)Ala-OH (1 equiv., 20 mg) in DMF (6 mL) at 0°C, EDC (1.1 equiv., 20 mg) and HOBt anhydrous (1.1 equiv., 14 mg) were added. After 10 min., NMM (1.1 equiv., 12 μL) and **7** as TFA salt (1.1 equiv.), were dissolved in the reaction mixture and stirred at 0°C for 10 min. After 24 hours at r.t., the reaction mixture was evaporated in rotary evaporator, the residue was dissolved in EtOAc and washed with 5% citric acid solution (3 times), $NaHCO_3$ s.s. (3 times) and NaCl s.s. (3 times). The organic layers were collected, dried over Na_2SO_4 anhydrous and filtered. The organic solvent was removed in rotary evaporator and high vacuum to obtain the crude product in 72% yield (eluent: EtOAc/MeOH = 9:1; R*f* = 0.5). The intermediate compound was deprotected with a mixture of TFA/DCM = 1:1 at room temperature for 1h, then was used as TFA salt for the following reaction without further modification.

^{1}H-NMR (300 MHz, $CDCl_3$) δ: 7.51 (d, 1H), 7.45 (d, 1H), 7.32-7.15 (m, 6H), 7.02 (d, 1H), 6.77 (d, 1H), 6.56 (m, 2H), 6.19 (m, 2H), 5.14 (t, 1H), 5.11 (d, 1H), 4.45 (m, 2H), 3.80 (t, 2H), 2.98 (q, 4H), 2.68 (m, 1H), 2.66 (q, 1H), 2.26 (s, 3H), 1.69 (m, 4H), 1.40 (s, 9H), 1.30 (d, 3H). LRMS (ESI) m/z 909.1 $[M+H]^+$.

Preparation of the final product 9. To a stirred solution of Boc-Tyr-OH (1 equiv., 15 mg) in DMF (6 mL) at 0°C, EDC (1.1 equiv., 10 mg) and HOBt anhydrous (1.1 equiv., 8 mg) were added. After 10 min., NMM (1.1 equiv., 5 μL) and **8** as TFA salt (1.1 equiv.), were dissolved in the reaction mixture and stirred at 0°C for 10 min. After 24 hours at r.t., the reaction mixture was evaporated in rotary evaporator, the residue was dissolved in EtOAc and washed with 5% citric acid solution (3

times), $NaHCO_3$ s.s. (3 times) and NaCl s.s. (3 times). The organic layers were collected, dried over Na_2SO_4 anhydrous and filtered. The organic solvent was removed in rotary evaporator and high vacuum to obtain the crude product as a white powder. The crude product was purified on silica gel column (eluent: EtOAc/MeOH = 9.8:0.2; R*f* = 0.3) to obtain the intermediate Boc-protected compound as a white powder in 37% yield. ^{1}H-NMR (300 MHz, $CDCl_3$) δ: 7.74 (d, 1H), 7.51 (d, 1H), 7.45 (d, 1H), 7.32-7.15 (m, 6H), 7.02 (d, 1H), 6.93-6.86 (m, 3H), 6.77 (d, 1H), 6.68 (dd, 2H), 6.56 (m, 2H), 6.19 (m, 2H), 5.14 (t, 1H), 5.11 (d, 1H), 4.45 (m, 2H), 4.33 (m, 1H), 3.80 (t, 2H), 2.98 (q, 4H), 2.68 (m, 1H), 2.66 (q, 1H), 2.48 (m, 1H), 2.26 (s, 3H), 1.69 (m, 4H), 1.40 (s, 9H), 1.30 (d, 3H). LRMS (ESI) m/z 1072.5 $[M+H]^+$.

The intermediate compound was deprotected with a mixture of TFA/DCM = 1:1 at room temperature for 1h; then it was purified in RP-HPLC (Column Water X-Bridge C18 bonded 4.6 x 150 mm, at a flow rate of 4 mL/min using as eluent a gradient of H_2O/acetonitrile 0.1% TFA starting from 5% acetonitrile to 90% acetonitrile in 40min, 4.0 mL/min, 20°C, 254 nm) to obtain the final product **9**. The purity of compound **9** was determined by analytical RP-HPLC and was found to be ≥ 96% (R_t 19.97 min; linear gradient 5% to 95% acetonitrile in 32 min) and LRMS (ESI): $[M+H^+]$ = 972.5 m/z.

^{1}H-NMR (300 MHz, DMSO-d_6) δ: 9.35 (s, 1H), 8.58 (d, 1H), 8.24-8.01 (m, 6H), 7.81 (d, 1H), 7.74 (t, 1H), 7.55 (m, 1H), 7.21 (m, 5H), 7.02 (d, 2H), 6.76 (s, 1H), 6.70 (d, 2H), 6.12 (s, 1H), 4.93 (m, 1H), 4.28 (t, 1H), 3.88 (m, 1H), 3.68 (m, 1H), 3.63 (t, 2H), 2.91-2.70 (m, 8H), 2.11 (s, 3H), 1.05 (d, 3H).

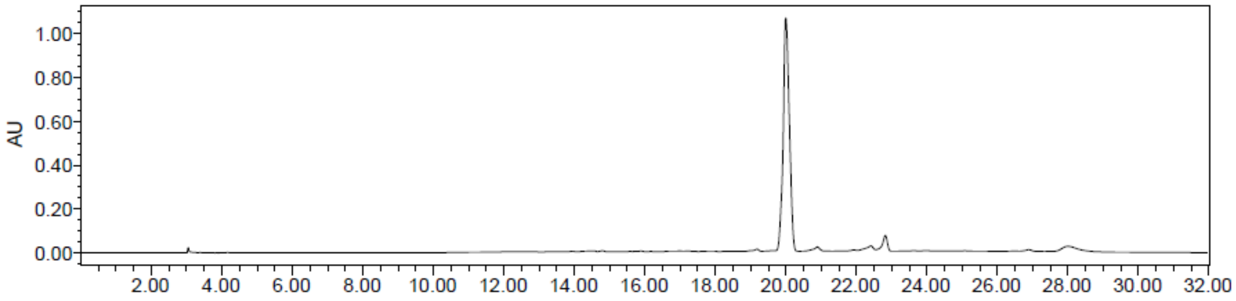

Peak	Retention time (min)	AREA%
**1**	19.97	96.00
**2**	22.82	4.00

In vitro binding assays

Chemicals

Tris-HCl, EGTA, NaCl, $MgCl_2$ x $6H_2O$, GDP, the GTP analogue GTPγS, were purchased from Sigma-Aldrich (Budapest, Hungary). Fatty acid free bovine serum albumin (BSA) was purchased from Serva (Heidelberg, Germany). Naloxone was kindly provided by the company Endo Laboratories DuPont de Nemours (Wilmington, DE, USA). HS665 was provided by Schmidhammer's group (University of Innsbruck, Austria). WIN55,212-2 and Rimonabant was purchased from Tocris Bioscience (Bristol, UK). The Rimonabant analogues **1**, **2**, **3**, **4**, **5**, opioid peptide **10** and the bivalent compound **9** were provided by Mollica's group. Ligands were dissolved in water with the exception of WIN55,212-2, Rimonabant and HS665, which were dissolved in DMSO. All compounds were stored in 1 mM stock solution at -20˚C. The radiolabelled GTP analogue, [^{35}S]GTPγS (specific activity: 1000 Ci/mmol) were purchased from Hartmann Analytic (Braunschweig, Germany). [^{3}H]DAMGO (specific activity: 38.8 Ci/mmol),[1,2] [^{3}H]IleDelt II (specific activity: 19,6 Ci/mmol), [^{3}H]WIN55,212-2 (specific activity: 13,1 Ci/mmol) and [^{3}H]HS665 (specific activity: 36.62 Ci/mmol),[3] were radiolabeled by the Laboratory of Chemical Biology group in BRC (Szeged, Hungary).

Animals

For membrane homogenate preparations male and female Wistar rats (250-300 g body weight) and male guinea pigs (~700 g body weight, LAL/HA/BR strain) were used. Rats were housed in the local animal house of BRC (Szeged, Hungary), while guinea pigs were housed in LAB-ÁLL Bt. (Budapest, Hungary). All the animals were kept in a temperature controlled room (21-24°C) under a 12:12 light and dark cycle and were provided with water and food *ad libitum*. All housing and experiments were conducted in accordance with the European Communities Council Directives (2010/63/EU) and the Hungarian Act for the Protection of Animals in Research (XXVIII.tv. 32.§). The total number of animals as well as their suffering was minimized.

Rat and guinea pig brain membrane homogenate preparation for binding assays

Rats and guinea pigs were decapitated and their brains were quickly removed. The brains were prepared for membrane preparation as previously described,[4] and partly used for binding experiments and partly were further prepared for the [^{35}S]GTPγS binding experiments. Briefly the brains were homogenized, centrifuged in ice-cold 50 mM Tris-HCl (pH 7.4) buffer and incubated at 37°C for 30 min in a shaking water-bath. After incubation the centrifugation was repeated as described above and the final pellet was suspended in 50 mM Tris-HCl pH 7.4 buffer containing 0.32 M sucrose, and stored at -80°C. For the [^{35}S]GTPγS binding experiments the final pellet of rat

brain membrane homogenate was suspended in ice-cold TEM (Tris-HCl, EGTA, $MgCl_2$) and stored at -80°C for further use.

Radioligand competition binding experiments

Aliquots of frozen rat brain membrane homogenates were centrifuged (18 000 rpm, 20 min, 4˚C) to remove sucrose and the pellets were suspended in 50 mM Tris-HCl buffer (pH 7.4). Membranes containing 0.4-0.8 mg/mL of protein were incubated in a final volume of 1 mL in the presence of unlabeled **5**, **10** and **9** in increasing concentrations (10^{-11}-10^{-5} M) in the presence of the appropriate radioligand in ~1 nM concentrations at certain temperature and amount of time depending on the radioligand ([^{3}H]DAMGO and [^{3}H]IleDelt II: 35˚C for 45 min; [^{3}H]HS665: 30˚C, 45 min; [^{3}H]WIN55,212-2: 30˚C for 60 min). Experiments performed with [^{3}H]HS665 where incubated together with guinea pig whole brain membrane homogenates (the guinea pig brain has significantly more kappa receptors than the rat brain), while the rest of the radioligands were incubated together with rat whole brain membrane homogenates. Additionally, in experiments performed with [^{3}H]WIN55,212-2 Rimonabant analogues, Rimonabant was also added to the incubation mixture in increasing concentrations (10^{-11}-10^{-5} M) together with 50 mM Tris/HCl, 2.5 mM EGTA, 5 mM $MgCl_2$ and 0.5 mg/mL fatty acid free BSA (pH 7.4). The level of non-specific binding was determined in the presence of 10 μM unlabelled naloxone or HS665 or WIN55,212-2, while total binding was determined in the absence of unlabeled compounds. The reaction was terminated by rapid filtration under vacuum (Brandel M24R Cell Harvester), and washed three times with 5 mL ice-cold 50 mM Tris-HCl or 50 mM Tris/HCl, 2.5 mM EGTA, 5 mM $MgCl_2$, 0.5 % BSA (pH 7.4) in case of [^{3}H]WIN55,212-2. The filtration was accomplished through Whatman GF/C ([^{3}H]DAMGO, [^{3}H]IleDelt II and [^{3}H]HS665) or GF/B ([^{3}H]WIN55,212-2) glass fibers. In case of [^{3}H]WIN55,212-2 the filter was presoaked in 0.1% polyethyleneimine 30 min before the filtration. The radioactivity of the filters was detected in UltimaGold™ MV aqueous scintillation cocktail with Packard Tricarb 2300TR liquid scintillation counter. The competition binding assays were performed in duplicate and repeated at least three times.

Table 1S. Affinity values of Rimonabant and its analogues, together with **9** and **10** ($logIC_{50}$ ± S.E.M. and K_i) in competition binding experiments on the cannabinoid receptors (CBR) and three opioid receptors with the indicated receptor specific radioligands. The opioid receptor selectivity ratios of **9** and **10** is also indicated. The values were determined according to the binding curves in Figure 2 as described in the 'Data analysis' section.

	**$logIC_{50}$ ± S.E.M. (M) (K_i)**				**Selectivity ratios**		
**Compounds**	**[^{3}H]WIN55,212[a] (CBR)**	**[^{3}H]DAMGO[a] (MOR)**	**[^{3}H]IleDelt II[a] (DOR)**	**[^{3}H]HS665[b] (KOR)**	**DOR/ MOR**	**KOR/ MOR**	**DOR/ KOR**
**Rimonabant**	-7.53 ± 0.08 (25 nM)	n.d.	n.d.	n.d.	-		
**1**	-6.71 ± 0.09 (125.9 nM)	n.d.	n.d.	n.d.	-		
**2**	-5.96 ± 0.09 (709.7 nM)	n.d.	n.d.	n.d.	-		
**3**	-6.53 ± 0.07 (192.9 nM)	n.d.	n.d.	n.d.	-		
**4**	-6.24 ± 0.1 (370.3 nM)	n.d.	n.d.	n.d.	-		
**5**	ambiguous[1]	ambiguous[1]	ambiguous[1]	-4.57 ± 1.26 (17.4 μM)	-		
**9**	-5.22 ± 0.33 (5.2 μM)	-6.69 ± 0.10 (34.4 nM)	-5.89 ± 0.10 (443.4 nM)	-6.58 ± 0.08 (169.6 nM)	12.88	4.93	2.61
**10**	ambiguous[1]	-8.21 ± 0.09 (1 nM)	-6.5 ± 0.1 (106.8 nM)	-6.24 ± 0.16 (368.6 nM)	106.8	368.6	0.28

[a]: performed in rat whole brain membrane homogenates

[b]: performed in guinea pig whole brain membrane homogenates

[1]: since the compound did not alter significantly the total specific binding of the radioligand, thus $logIC_{50}$ and K_i values cannot be interpreted

n.d.: not determined

Functional [^{35}S]GTPγS binding experiments

The functional [^{35}S]GTPγS binding experiments were performed as previously described,[5,6] with modifications. Briefly the rat brain membrane homogenates containing ~10 μg/mL protein were incubated at 30°C for 60 min in Tris-EGTA buffer (pH 7.4) composed of 50 mM Tris-HCl, 1 mM EGTA, 3 mM $MgCl_2$, 100 mM NaCl, containing 20 MBq/0.05 cm^3 [^{35}S]GTPγS (0.05 nM) and increasing concentrations (10^{-10}-10^{-5} M) of Rimonabant analogues, opioid peptide **10** and bivalent compound **9** together with excess GDP (30 μM) in a final volume of 1 mL. Total binding was measured in the absence of test compounds, non-specific binding was determined in the presence of 10 μM unlabelled GTPγS. The bound and unbound [^{35}S]GTPγS was separated as described in the

competition binding assays section through Whatmann GF/B glass fibers. The radioactivity of the filters was also detected as described above. [^{35}S]GTPγS binding experiments were performed in triplicates and repeated at least three times.

Data analysis

The specific binding of all radiolabeled compounds was calculated by the subtraction of non-specific binding from total binding and was given in percentage. The data was normalized to total specific binding, which was settled 100%, which in case of [^{35}S]GTPγS also represents the basal activity of the G-protein. Experimental data were presented as means ± S.E.M in the function of the applied ligand concentration range in logarithm form. Points were fitted with the professional curve fitting program, GraphPad Prism 5.0 (GraphPad Prism Software Inc., San Diego, CA), using non-linear regression. In the radioligand competition binding assays the 'One-site competition', while in [^{35}S]GTPγS binding assays the 'Sigmoid dose-response' equation was applied to determine IC_{50} (unlabeled ligand affinity) and the maximum G-protein efficacy (E_{max}) and ligand potency (EC_{50}), respectively. For IC_{50} and EC_{50} values standard error is only given in their logarithm form by the curve fitting program due to the data representation. In competition binding experiments the K_i value was calculated according to Cheng-Prusoff equation.[7]

Figure 1S. The effect of Rimonabant analogues and Rimonabant (**A**), **9** and **10** (**B**) on G-protein activity in [^{35}S]GTPγS binding assays. In figure B, **5** is indicated again for better comparison. Figures represents the specific binding of [^{35}S]GTPγS in percentage in the presence of increasing concentrations (10^{-10}-10^{-5} M) of the indicated ligands in rat brain membrane homogenates. "Basal" on the x-axis indicates the basal activity of the monitored G-protein, which is measured in the absence of the compounds and also represents the total specific binding of [^{35}S]GTPγS. The level of basal activity was defined as 100% and is presented with a dotted line. Points represent means ± S.E.M. for at least three experiments performed in triplicate.

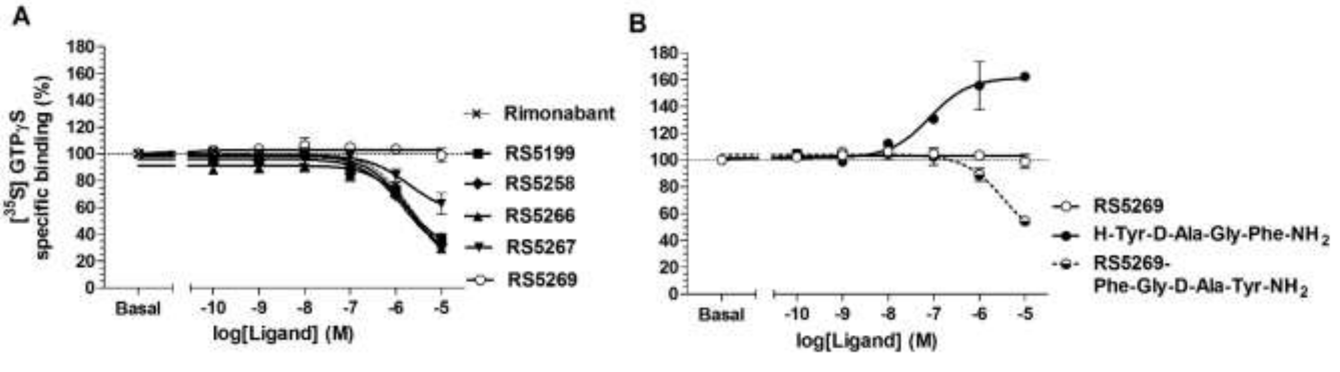

Legend	
RS5199	**1**
RS5258	**2**
RS5267	**3**
RS5266	**4**
RS5269	**5**

RS5269-Phe-Gly-D-Ala-TyrNH_2	**9**
Tyr-D-Ala-Gly-Phe-NH_2	**10**

In vivo nociception tests

Animals

Male CD-1 mice (Harlan, Italy) weighting 25-30 g were used for all experiments. Mice were housed for at least 1 week before the experimental sessions in colony cages (7 mice in each cage) under standard light (from 7.00 a.m. to 7.00 p.m.), temperature (21±1°C), relative humidity (60±10%) with food and water available ad libitum. The research protocol was approved by the Service for Biotechnology and Animal Welfare of the Istituto Superiore di Sanità and authorized by the Italian Ministry of Health, according to Legislative Decree 26/14, which implemented the European Directive 2010/63/UE on laboratory animal protection in Italy. Animal welfare was routinely checked by veterinarians from the Service for Biotechnology and Animal Welfare. The authors further ensure that all efforts were made to minimize animal suffering and the number used.

Surgery for intracerebroventricular (i.c.v.) injections

For i.c.v. injections, mice were lightly anesthetized with isoflurane, and an incision was made in the scalp. Injections were performed using a 10 μL Hamilton microsyringe at a point 2-mm caudal and 2-mm lateral from the bregma at a depth of 3 mm in a volume of 5 μL as previously described.[8]

Hot plate and tail flick test

In the case of hot plate test, the thermal nociception was assessed with a commercially available apparatus consisting in a metal plate 25x25 cm (Ugo Basile, Italy) heated to a constant temperature of 55±0.1°C, on which a plastic cylinder (20 cm diameter, 18 cm high) was placed. The time of latency (s) was recorded from the moment the animal was inserted inside the cylinder up to when it licked its paws or jerked them off the hot plate; jumped off the hot plate; the latency exceeded the cut-off time of 60 s. The baseline was calculated as mean of three readings recorded before testing at intervals of 15-30 min. The time course of latency was then determined at 15, 30, 45, 60, and 90 min after treatment. The tail flick latency was obtained using a commercial unit (Ugo Basile, Italy), consisting of an infrared radiant light source (100 W, 15 V bulb) focused onto a photocell utilizing an aluminum parabolic mirror. During the trials the mice were gently hand-restrained with a glove. Radiant heat was focused 3-4 cm from the tip of the tail, and the latency (s) of the tail withdrawal recorded. The measurement was interrupted if the latency exceeded the cut off time (15 s at 15 V). Also in this case, the baseline was calculated as mean of three readings recorded before testing at intervals of 15-30 min and the time course of latency determined at 15, 30, 45, 60, and 90 min after treatment. In both the hot plate and tail flick tests, data were expressed as time course of the percentage of maximum effect (%MPE)=(post drug latency – baseline latency)/(cut-off time – baseline latency) x 100. On each test day compound solutions were freshly prepared using DMSO:0.9% saline 1:5 v/v. ~~Compound under investigation was~~ These solutions were injected at doses of 0, 1, 5 and 10 μg/mouse in a volume of 5 μL/mouse. The significance among the groups

was evaluated with the analysis of variance followed by Dunnett's post-hoc comparisons using GraphPad Prism 6.03 software. Statistical significance was assumed at least for $P<0.05$.

In vitro hypothalamic perfusion test

Hypothalamic synaptosomes were obtained from 18 male adult Sprague-Dawley rats (290-340 g), as previously described.[9]

Then, the synaptosome suspension was incubated at 37°C, under O_2/CO_2 95%/5%, pH 7.35-7.45, in Krebs-Ringer buffer (mM: NaCl 125, KCl 3, $MgSO_4$ 1.2, $CaCl_2$ 1.2, Tris-HCl 10, glucose 10, ascorbic acid 1). After the incubation period, identical aliquots of synaptosome suspension (1.24 mg protein determined by bicinchoninic acid protein assay) were layered onto 0.8 µm Millipore filters, placed into 37°C water-jacketed superfusion chambers [18 different chambers for each experiment and perfused with Krebs-Ringer buffer (0.6 mL/min)], and perfusate was collected (2 min fractions) to detect released neurotransmitters by HPLC coupled to electrochemical detection. The analytical conditions for amine identification and quantification in the perfusate fractions were selected on the basis of previous experiments performed on hypothalamus homogenate.[10]

The experimental protocol includes three phases: 10 min perfusion with Krebs-Ringer buffer in basal condition (pre-stimulus); 10 min perfusion with the peptide (10 µM) added to Krebs-Ringer buffer (stimulus); 8 min perfusion with Krebs-Ringer buffer for return to basal condition (post-stimulus). Perfusate was collected starting from the 8th minute of pre-stimulus. Perfusate aliquots were stored at -80°C, then lyophilized and finally suspended in 1 mL HPLC grade water for neurotransmitter quantification. Neurotransmitter release was expressed as mean percentage release recovered in the stimulus and post-stimulus, compared with initial basal conditions (pre-stimulus period).

ADMET in silico profiling

Molecular datasets used by Zhao[11] and Shen[12] were compiled and merged into one single dataset of 2845 unique molecules with known capabilities to overcame or not the BBB. Two classification models were built using Random Forest,[13] and Logistic regression,[14] statistics applied on calculated circular fingerprints with 2 layers and 4096 bits. The new classification models were comparable with those previously reported and were characterized by very good statistical values (Table 2S).

Table 2S. Random forest and Logistic regression derived model for BBB permeability and compounds **5**, **9** and **10** predictions

**Model**[a]	**Accuracy**[b]	**MCC**[c]	**ROC AUC**[d]	**P-R AUC**[e]	**Compounds BBB permeability predictions**					
					**5**		**9**		**10**	
					**BBB-**[f]	**BBB+**[g]	**BBB-**[e]	**BBB+**[f]	**BBB-**[e]	**BBB+**[f]
Random Forest	0.94	0.83	0.96	0.99	0.29	0.71	0.62	0.38	0.55	0.45
Logistic Regression	0.95	0.86	0.97	0.99	0.01	0.99	0.75	0.25	0.80	0.20

a: Models were derived and validated trough 100 iterations cross-validation using two groups.

b: The Accuracy classification score. Accuracy = (TP + TN) / (TP + FP + FN + TN), where TP = True Positives and TN = True Negatives.

c: The Matthews correlation coefficient. It takes into account true and false positives and negatives and is generally regarded as a balanced measure which can be used even if the classes are of very different sizes. The MCC is in essence a correlation coefficient value between -1 and +1. A coefficient of +1 represents a perfect prediction, 0 an average random prediction and -1 an inverse prediction.

MCC = (TP * TN – FP * FN) / ((TP + FP) * (TP + FN) * (TN + FP) * (TN + FN))^0.5

d: Receiver Operating Characteristic Area Under the Curve. The curve is created by plotting the true positive rate (TPR/Recall) against the false positive rate (FPR/Fall-out) at various threshold settings.

e: Precision-recall AUC. This score corresponds to the area under the Precision-Recall curve. The curve is created by plotting the true positive rate (TPR/Recall) against the positive predictive value (PPV/Precision) at various threshold settings.

f: A compound is flagged as BBB- if cannot penetrate the blood-brain barrier

g: A compound is flagged as BBB+ if can penetrate the blood-brain barrier

Table 3S. AdmetSAR classification derived data for diverse chemicals associated with known Absorption, Distribution, Metabolism, Excretion and Toxicity profiles for compounds **5**, **9** and **10**.

	Model	5		9		10	
		Result	Probability	Result	Probability	Result	Probability
Absorption	Blood-Brain Barrier	BBB+	0.8006	BBB-	0.8223	BBB-	0.6971
	Human Intestinal Absorption	HIA+	0.9943	HIA+	0.9904	HIA+	0.9295
	Caco-2 Permeability	Caco2-	0.6642	Caco2-	0.6951	Caco2-	0.8659
	P-glycoprotein Substrate	Substrate	0.6926	Substrate	0.7862	Substrate	0.7058
	P-glycoprotein Inhibitor	Non-inhibitor	0.6889	Non-inhibitor	0.8361	Non-inhibitor	0.9329
		Non-inhibitor	0.6859	Non-inhibitor	0.6110	Non-inhibitor	0.9550
	Renal Organic Cation Transporter	Non-inhibitor	0.6373	Non-inhibitor	0.8091	Non-inhibitor	0.8609
Metabolism	CYP450 2C9 Substrate	Non-substrate	0.8051	Non-substrate	0.8530	Non-substrate	0.8077
	CYP450 2D6 Substrate	Non-substrate	0.7241	Non-substrate	0.7786	Non-substrate	0.7761
	CYP450 3A4 Substrate	Substrate	0.6413	Substrate	0.6706	Non-substrate	0.5504
	CYP450 1A2 Inhibitor	Non-inhibitor	0.5923	Non-inhibitor	0.9469	Non-inhibitor	0.9275
	CYP450 2C9 Inhibitor	Non-inhibitor	0.5702	Non-inhibitor	0.7210	Non-inhibitor	0.8807
	CYP450 2D6 Inhibitor	Non-inhibitor	0.6376	Non-inhibitor	0.8489	Non-inhibitor	0.8858
	CYP450 2C19 Inhibitor	Inhibitor	0.5418	Non-inhibitor	0.8191	Non-inhibitor	0.8283
	CYP450 3A4 Inhibitor	Non-inhibitor	0.8429	Non-inhibitor	0.7530	Non-inhibitor	0.7215
	CYP Inhibitory Promiscuity	High CYP Inhibitory Promiscuity	0.7208	Low CYP Inhibitory Promiscuity	0.8407	Low CYP Inhibitory Promiscuity	0.9120
Toxicity	Human Ether-a-go-go-Related Gene Inhibition	Weak inhibitor	0.8008	Weak inhibitor	0.7323	Weak inhibitor	0.9773
		Inhibitor	0.9234	Inhibitor	0.7517	Non-inhibitor	0.8104
	AMES Toxicity	Non AMES toxic	0.5677	Non AMES toxic	0.5560	Non AMES toxic	0.8231
	Carcinogens	Non-carcinogens	0.7363	Non-carcinogens	0.6157	Non-carcinogens	0.8695
	Fish Toxicity	High FHMT	0.9271	High FHMT	0.9831	High FHMT	0.6440
	Tetrahymena Pyriformis Toxicity	High TPT	0.9204	High TPT	0.9699	High TPT	0.9211
	Honey Bee Toxicity	Low HBT	0.9344	Low HBT	0.9272	Low HBT	0.8028
	Biodegradation	Not ready biodegradable	0.9790	Not ready biodegradable	1.0000	Not ready biodegradable	0.9454
	Acute Oral Toxicity	III	0.5992	III	0.5885	III	0.7369
	Carcinogenicity (Three-class)	Non-required	0.4950	Non-required	0.5358	Non-required	0.6641

Table 4S. Calculated drug likeness properties for compounds **5, 9** and **10** by Molinspiration web server (http://www.molinspiration.com/).

	ADMET Features	5	9	10
Drug Likeness Properties	miLogP[a]	0.93	3.64	-0.27
	TPSA[b]	68.46	205.71	176.64
	Natoms[c]	28	60	33
	MW[d]	419.34	856.81	455.51
	nON[e]	6	15	10
	nOHNH[f]	3	7	8
	N violations[g]	0	3	1
	Nrotb[h]	4	15	11
	Volume[i]	355.03	747.15	416.64

a: LogP (octanol/water partition coefficient). LogP is calculated by the methodology developed by Molinspiration as a sum of fragment-based contributions and correction factors. Method is very robust and is able to process practically all organic, and most organometallic molecules.

b: Molecular Polar Surface Area TPSA is calculated based on the methodology published by Ertl *et al.*[15] as a sum of fragment contributions. O- and N- centered polar fragments are considered. PSA has been shown to be a very good descriptor characterizing drug absorption, including intestinal absorption, bioavailability, Caco-2 permeability and blood-brain barrier penetration.

c: Number of heavy atoms.

d: Molecular Volume. Method for calculation of molecule volume developed at Molinspiration is based on group contributions. These have been obtained by fitting sum of fragment contributions to "real" 3D volume for a training set of about twelve thousand, mostly drug-like molecules. 3D molecular geometries for a training set were fully optimized by the semiempirical AM1 method.

e: Number of hydrogens atoms on oxygens and nitrogens

f: Number of oxygen and nitrogen atoms

g: Lipinsky rule of 5 properties,[16] is set of simple molecular descriptors used by Lipinski in formulating his "Rule of 5". The rule states, that most "drug-like" molecules have logP <= 5, molecular weight <= 500, number of hydrogen bond acceptors <= 10, and number of hydrogen bond donors <= 5. Molecules violating more than one of these rules may have problems with bioavailability. In red are reported Lipinski rules violations

h: nrotb, this simple topological parameter is a measure of molecular flexibility. It has been shown to be a very good descriptor of oral bioavailability of drugs. Rotatable bond is defined as any single non-ring bond, bounded to nonterminal heavy (*i.e.* non-hydrogen) atom. Amide C-N bonds are not considered because of their high rotational energy barrier.

i: Molecular volume.

Table 5S. Classification models for BBB+/BBB- permeability.[17]

<table>
<tr><th>FP[a]</th><th>Threshold[b]</th><th>AdaBoost[c]</th><th>SVM[d]</th></tr>
<tr><td>MACCS</td><td>0.02</td><td>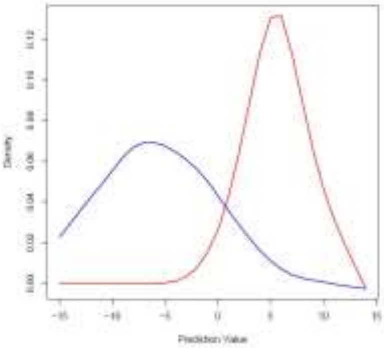
</td><td>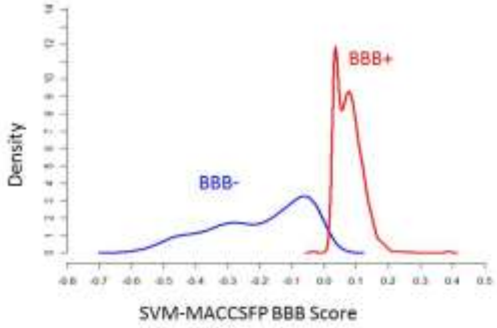
</td></tr>
<tr><td>Molprint</td><td>0.00</td><td>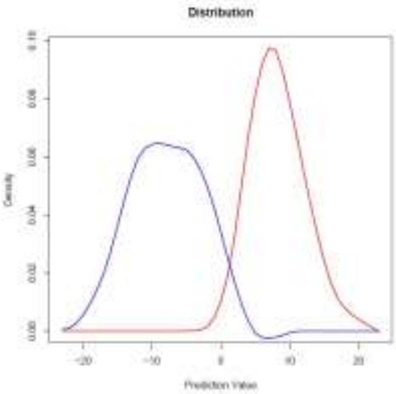
</td><td>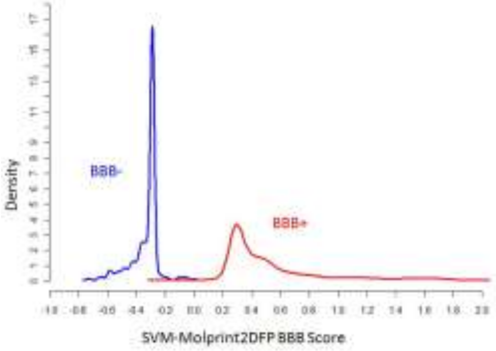
</td></tr>
<tr><td>Openbabel</td><td>0.00</td><td>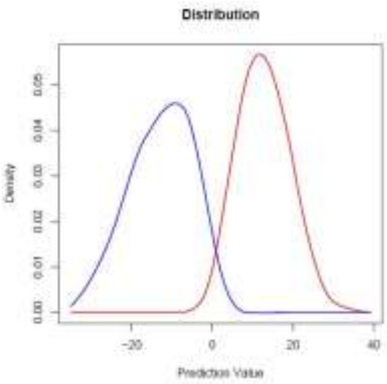
</td><td>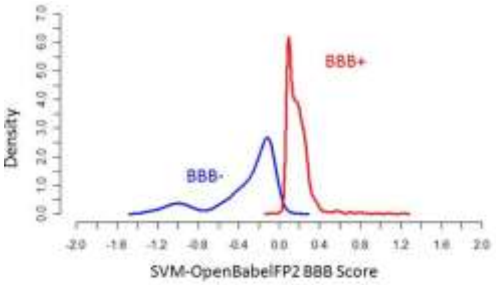
</td></tr>
<tr><td>PubChem</td><td>0.00</td><td>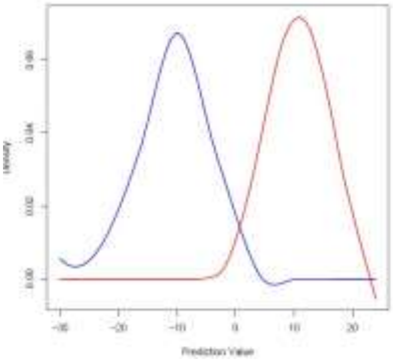
</td><td>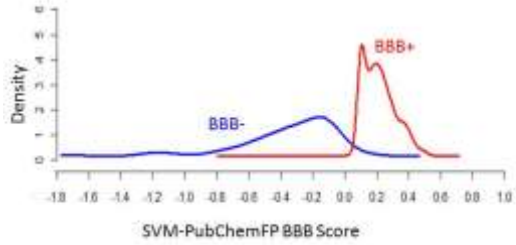
</td></tr>
</table>

a: molecular fingerprints calculations method; b: BBB-/BBB+ permeability; c: Adaptive Boosting machine learning meta-algorithm classification method graphical profile (blu line is for BBB-, red line is for BBB+); d: support vector machines supervised learning models with associated learning algorithms classification method graphical profile (blu

line is for BBB-, red line is for BBB+).

Table 6S. Compounds **5**, **9** and **10** BBB permeabilities evaluation by molecular fingerprints models as calculated by BBB online predictor (www.cbligand.org/BBB).[17] For interpretation see Table 5S.

Compound	AdaBoost				SVM			
	MACCS	Molprint	Openbabel	PubChem	MACCS	Molprint	Openbabel	PubChem
**5**	2.587	5.741	18.621	15.758	0.023	0.378	0.199	0.076
**9**	-0.409	-1.796	1.227	-4.571	-0.01	-0.052	0.061	-0.092
**10**	-0.397	-1.346	-3.114	-10.757	-0.01	-0.022	-0.078	-0.167

Figure 2S. Similarity maps for compounds **5**, **9** and **10**. Color scheme: removing bits decreases BBB permeability (*i.e.* positive difference) (green), no change in BBB permeability (gray), removing bits increases BBB permeability (*i.e.* negative difference) (pink). The bit vectors of the circular fingerprints had the size 4096 bits.

Compd	Random Forest	Logistic Regression
**5**	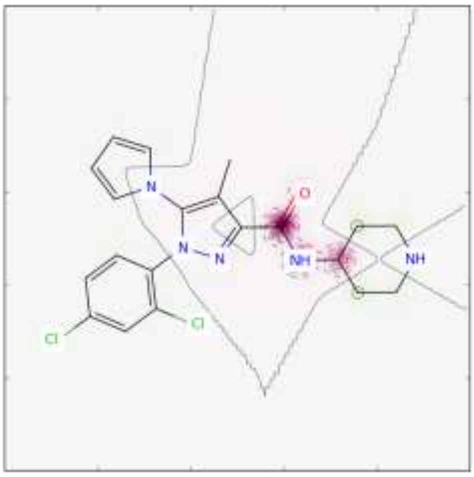	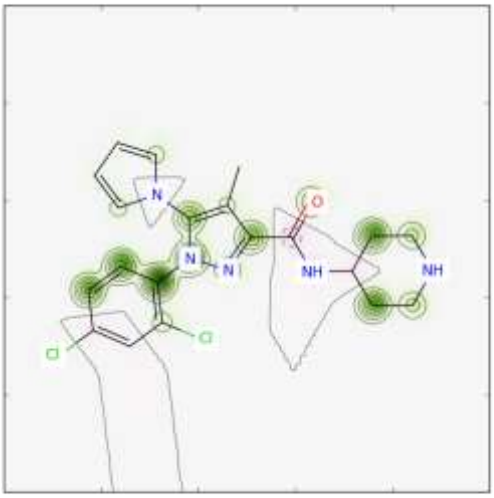
**9**	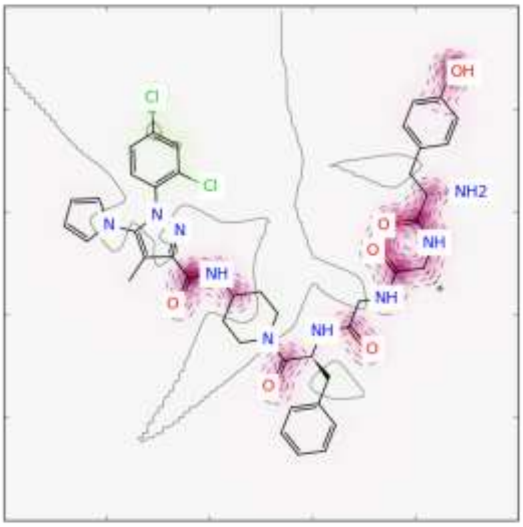	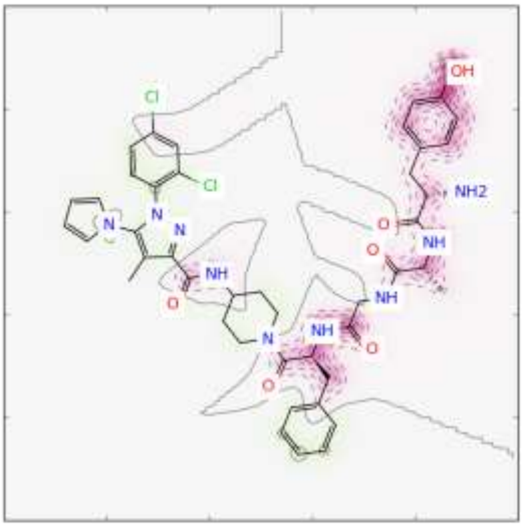
**10**	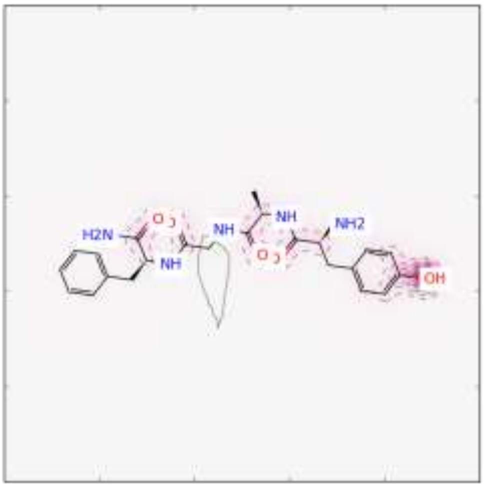	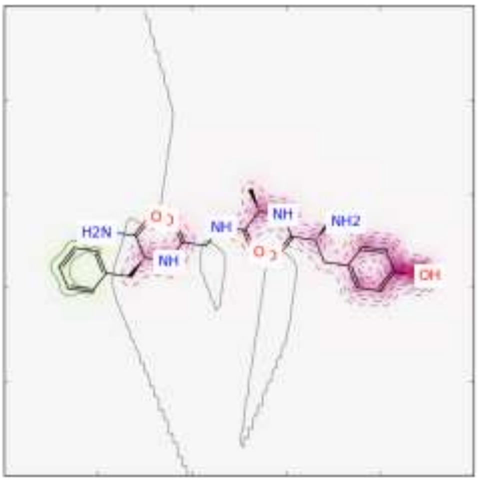

References

(1) Silvestri R, Cascio MG, La Regina G, Piscitelli F, Lavecchia A, Brizzi A, Pasquini S, Botta M, Novellino E, Di Marzo V, Corelli F. Synthesis, cannabinoid receptor affinity, and molecular modeling studies of substituted 1-aryl-5-(1H-pyrrol-1-yl)-1H-pyrazole-3-carboxamides. J. Med. Chem. **2008;**51:1560-1576.

(2) Ioja E, Tourwé D, Kertész I, Tóth G, Borsodi A, Benyhe S. Novel diastereomeric opioid tetrapeptides exhibit differing pharmacological activity profiles. Brain Res. Bull. **2007;**74:119-129.

(3) Guerrieri E, Mallareddy JR, Tóth G, Schmidhammer H, Spetea M. Synthesis and pharmacological evaluation of [(3)H]HS665, a novel, highly selective radioligand for the kappa opioid receptor. ACS Chem. Neurosci. **2015;**6:456-463.

(4) Benyhe S, Farkas J, Tóth G, Wollemann M. Met5-enkephalin-Arg6-Phe7, an endogenous neuropeptide, binds to multiple opioid and nonopioid sites in rat brain. J. Neurosci. Res. **1997;**48:249-258.

(5) Sim LJ, Selley DE, Childers SR. In vitro autoradiography of receptor-activated G proteins in rat brain by agonist-stimulated guanylyl 5'-[gamma-[35S]thio]-triphosphate binding. Proc Natl Acad Sci USA **1995;**92:7242-7246.

(6) Traynor J, Nahorski S. Modulation by μ-opioid agonists of guanosine-5'-O-(3- thio) triphosphate binding to membranes from human neuroblastoma SH-SY5Y cells. Mol. Pharmacol. **1995;**47:848-854.

(7) Cheng Y; Prusoff WH. Relationship between the inhibition constant (K1) and the concentration of inhibitor which causes 50 per cent inhibition (I50) of an enzymatic reaction. Biochem. Pharmacol. **1975;**22:3099-3108.

(8) Pieretti S, Di Giannuario A, De Felice M, Perretti M, Cirino G. Stimulus-dependent specificity for annexin 1 inhibition of the inflammatory nociceptive response: the involvement of the receptor for formylated peptides. Pain **2004;**109:52-63.

(9) Brunetti L, Recinella L, Di Nisio C, Chiavaroli A, Leone S, Ferrante C, Orlando G, Vacca M. Effects of visfatin/PBEF/NAMPT on feeding behaviour and hypothalamic neuromodulators in the rat. J. Biol. Regul. Homeost. Agents **2012;**26:295-302.

(10) Brunetti, L.; Orlando, G.; Ferrante, C.; Recinella, L.; Leone, S.; Chiavaroli, A.; Di Nisio, C.; Shohreh, R.; Manippa, F.; Ricciuti, A.; Vacca, M. Orexigenic effects of omentin-1 related to decreased CART and CRH gene expression and increased norepinephrine synthesis and release in the hypothalamus. *Peptides* **2013**, *44*, 66-74.

(11) Zhao YH, Abraham MH, Ibrahim A, Fish PV, Cole S, Lewis ML, de Groot MJ, Reynolds DP. Predicting penetration across the blood-brain barrier from simple descriptors and fragmentation schemes. J Chem Inf Model **2007;**47:170-175.

(12) Shen J, Cheng F, Xu Y, Li W, Tang Y. Estimation of ADME properties with substructure pattern recognition. J Chem Inf Model **2010;**50:1034-1041.

(13) Breiman L. Random Forests. *Machine Learning* **2001;**45:5-32.

(14) Hastie T, Tibshirani R, Friedman J. The Elements of Statistical Learning. Springer New York Inc.: **2001**.

(15) Ertl P, Rohde B, Selzer P. Fast calculation of molecular polar surface area as a sum of fragment-based contributions and its application to the prediction of drug transport properties. J Med Chem **2000;**43:3714-3717.
(16) Lipinski CA, Lombardo F, Dominy BW, Feeney PJ. Experimental and computational approaches to estimate solubility and permeability in drug discovery and development settings. Adv Drug Deliv Rev **2001;**46:3-26.
(17) Liu H, Wang L, Lv M, Pei R, Li P, Pei Z, Wang Y, Su W, Xie XQ. AlzPlatform: an Alzheimer's disease domain-specific chemogenomics knowledgebase for polypharmacology and target identification research. J Chem Inf Model **2014;**54:1050-1060.
